# A randomized phase 2 study of sapanisertib in combination with paclitaxel versus paclitaxel alone in women with advanced, recurrent, or persistent endometrial cancer

**DOI:** 10.1016/j.ygyno.2023.09.013

**Published:** 2023-10-14

**Authors:** Sileny N. Han, Amit Oza, Nicoletta Colombo, Ana Oaknin, Francesco Raspagliesi, Robert M. Wenham, Elena Ioana Braicu, Andrea Jewell, Vicky Makker, Jonathan Krell, Eva María Guerra Alía, Jean-François Baurain, Zhenqiang Su, Rachel Neuwirth, Sylvie Vincent, Farhad Sedarati, Douglas V. Faller, Giovanni Scambia

**Affiliations:** a Department of Obstetrics and Gynecology, University Hospitals Leuven, Leuven Cancer Institute, Leuven, Belgium; b Department of Oncology, KU Leuven, Gynaecological Oncology, University Hospitals Leuven, Leuven, Belgium; c Division of Medical Oncology & Hematology, Princess Margaret Cancer Centre, University Health Network, Toronto, Ontario, Canada; d Obstetrics and Gynaecology, University of Milano-Bicocca and European Institute of Oncology IRCCS, Milan, Italy; e Gynaecologic Cancer Programme, Vall d’Hebron Institute of Oncology (VHIO), Hospital Universitari Vall d’Hebron, Vall d’Hebron Barcelona Hospital Campus, Barcelona, Spain; f Gynecological Oncology Unit, Fondazione IRCCS Istituto Nazionale Tumori, Milan, Italy; g Department of Gynecologic Oncology, Moffitt Cancer Center, Tampa, FL, USA; h Department for Gynecology Campus Virchow, Charité Medical University Berlin, Berlin, Germany; i Obstetrics and Gynecology, University of Kansas Cancer Center, Kansas City, KS, USA; j Medical Oncology, Memorial Sloan Kettering Cancer Center, New York City, NY, USA; k Department of Medical Oncology, Imperial College London, London, UK; l Medical Oncology Department, Hospital Universitario Ramon y Cajal, Madrid, Spain; m Medical Oncology Department, Cliniques universitaires Saint-Luc, Université catholique de Louvain, Brussels, Belgium; n Computational Biology, Takeda Development Center Americas, Inc., Lexington, MA, USA; o Biostatistics, Takeda Development Center Americas, Inc., Lexington, MA, USA; p Translational Medicine, Takeda Development Center Americas, Inc., Lexington, MA, USA; q Oncology Clinical Research, Takeda Development Center Americas, Inc., Lexington, MA, USA; r Women, Children and Public Health Sciences, Fondazione Policlinico Universitario Agostino Gemelli IRCCS, Catholic University of the Sacred Heart School of Medicine, Rome, Italy

**Keywords:** Sapanisertib, Endometrial cancer, Advanced, Metastatic, Recurrent, Mammalian target of rapamycin complexes 1 and 2 inhibitor, Paclitaxel

## Abstract

**Objective.:**

This phase 2 study investigated sapanisertib (selective dual inhibitor of mTORC1/2) alone, or in combination with paclitaxel or TAK-117 (a selective small molecule inhibitor of PI3K), versus paclitaxel alone in advanced, recurrent, or persistent endometrial cancer.

**Methods.:**

Patients with histologic diagnosis of endometrial cancer (1–2 prior regimens) were randomized to 28-day cycles on four treatment arms: 1) weekly paclitaxel 80 mg/m^2^ (days 1, 8, and 15); 2) weekly paclitaxel 80 mg/m^2^ + oral sapanisertib 4 mg on days 2–4, 9–11, 16–18, and 23–25; 3) weekly sapanisertib 30 mg, or 4) sapanisertib 4 mg + TAK-117 200 mg on days 1–3, 8–10, 15–17, and 22–24.

**Results.:**

Of 241 patients randomized, 234 received treatment (paclitaxel, *n* = 87 [3 ongoing]; paclitaxel +sapanisertib, *n* = 86 [3 ongoing]; sapanisertib, *n* = 41; sapanisertib+TAK-117, *n* = 20). The sapanisertib and sapanisertib+TAK-117 arms were closed to enrollment after futility analyses. After a median follow-up of 14.4 (paclitaxel) versus 17.2 (paclitaxel+sapanisertib) months, median progression-free survival (PFS; primary endpoint) was 3.7 versus 5.6 months (hazard ratio [HR] 0.82; 95% confidence interval [CI] 0.58–1.15; *p* = 0.139); in patients with endometrioid histology (*n* = 116), median PFS was 3.3 versus 5.7 months (HR 0.66; 95% CI 0.43–1.03). Grade ≥ 3 treatment-emergent adverse event rates were 54.0% with paclitaxel versus 89.5% paclitaxel+sapanisertib.

**Conclusions.:**

Our findings support inclusion of chemotherapy combinations with investigational agents for advanced or metastatic disease. The primary endpoint was not met and toxicity was manageable.

**Trial registration:**

ClinicalTrials.gov number, NCT02725268

## 1. Introduction

Advanced or recurrent endometrial cancer is largely chemoresistant [1], and the prognosis for patients is poor [2], with a 5-year survival rate of 17% in patients with distant metastases [3]. In patients failing first-line chemotherapy, single-agent paclitaxel has demonstrated overall response rates (ORRs) of ~27% [4,5]. Novel agents and combinations have been investigated in phase 2/3 trials, and advancements in immunotherapy treatments have been made; however, chemotherapeutic treatment options remain limited [6–8].

Endometrial cancer is a heterogeneous disease with varying outcomes depending on histological tumor subtype and molecular alterations [9–11]. Endometrioid tumors are characterized by DNA polymerase epsilon catalytic subunit (POLE) mutations, microsatellite instability (MSI; impaired DNA mismatch repair of repeat DNA sequences of typically 1–6 base pairs [microsatellites]), and copy-number low/microsatellite stable (MSS) status [12]. The majority of low-grade endometrioid tumors have MSS status (60%) [13], which is associated with intermediate prognosis [11,14]. MSI is present in ~30% of recurrent endometrial tumors [15], and may correlate with survival [16], although an association between high MSI and prognosis has not consistently been demonstrated [17]. Approximately 25% of high-grade endometrioid tumors, and most non-endometrioid tumors, have copy-number high/“serous-like” characteristics [9,11], and typically have a poor prognosis [10]. Both endometrioid and non-endometrioid tumor histologies harbor mutations that upregulate the phosphoinositide-3 kinase/protein kinase B/mammalian target of rapamycin (PI3K/AKT/mTOR) pathway [18]. mTOR inhibitors are therefore a potential treatment option for advanced endometrial cancer.

First-generation mTOR inhibitors (rapalogs) that target mTOR complex 1 (mTORC1, e.g., temsirolimus and ridaforolimus), have demonstrated preliminary antitumor activity in small patient populations with recurrent endometrial cancer. However, isolated inhibition of mTORC1 without mTORC2 abrogates normal mTORC1-mediated feedback inhibition of insulin receptor substrate 1, enhancing downstream activity of AKT, which is suspected to play a role in the acquisition of treatment resistance [19]. Sapanisertib is an oral, potent, and highly selective inhibitor of mTOR kinase that exhibits dual specificity against mTORC1 and 2; this dual inhibition mitigates the feedback activation of AKT that facilitates resistance to rapalogs [20]. Sapanisertib has shown promising antitumor activity in preclinical studies, both alone and in combination with TAK-117 (serabelisib), a selective small molecule inhibitor of PI3K, or paclitaxel, in bladder cancer models [21]. We speculated that by targeting the PI3K/AKT/mTOR pathway through inhibition of mTORC1 and 2, sapanisertib could potentiate the cytotoxic effects of paclitaxel in patients with recurrent endometrial cancer. Furthermore, addition of PI3K inhibition may provide more robust inhibition of the PI3K/AKT/mTOR pathway.

This randomized, phase 2, 4-arm study assessed the efficacy and safety of sapanisertib alone, or in combination with paclitaxel or TAK-117, versus paclitaxel alone in women with advanced, recurrent, or persistent endometrial cancer.

## 2. Patients and methods

### 2.1. Patients

Female adults with a histologic diagnosis of advanced, recurrent, or persistent endometrial cancer (including endometrioid, serous, mixed adenocarcinoma, clear-cell carcinoma, or carcinosarcoma) that had progressed after prior platinum-based treatment were eligible. Patients were required to have at least 1 but no more than two prior platinum-based chemotherapeutic regimens for management of endometrial cancer. Patients were excluded if they had received previous treatment with any weekly taxane regimen, PI3K/mTOR, TORC1/2 inhibitors, were taking proton pump inhibitors (PPIs) within 7 days of the first dose of study drug, required treatment with PPIs throughout the trial, or were taking H_2_ receptor antagonists within 24 h of the first dose of study drug (see Supplementary Methods for eligibility criteria). The study was conducted according to the protocol, the ethical principles that have their origin in the Declaration of Helsinki, in accordance with International Conference for Harmonisation, and all applicable local regulations. All patients provided written informed consent.

### 2.2. Study design and oversight

This was a phase 2, open-label, multicenter study (NCT02725268) conducted across 60 study sites in North America, Europe, and Australia. Patients were randomized 1:1:1:1 to 28-day cycles on four treatment arms: 1) weekly single-agent paclitaxel 80 mg/m^2^ intravenously (days 1, 8, and 15); 2) weekly paclitaxel 80 mg/m^2^ in combination with sapanisertib 4 mg by mouth (PO) on days 2–4, 9–11, 16–18, and 23–25; 3) weekly single-agent sapanisertib 30 mg PO; or 4) sapanisertib 4 mg in combination with TAK-117 200 mg both dosed on days 1–3, 8–10, 15–17, and 22–24. Patients received treatments until unacceptable toxicity or disease progression. Patients were stratified (prespecified) by histological subtype (endometrioid vs non-endometrioid), lines of prior chemotherapy (1 vs 2), and prior taxane therapy other than a weekly regimen (yes vs no).

The primary endpoint was progression-free survival (PFS), assessed by the investigator according to the Response Evaluation Criteria in Solid Tumors, version 1.1; secondary efficacy endpoints included time to progression, overall survival (OS), ORR, clinical benefit rate (CBR; ORR + stable disease), CBR-16 (CBR at 16 weeks), and duration of response. PFS, time to progression, and OS were assessed from the time of randomization. The incidence of treatment-emergent adverse events (TEAEs) was a secondary safety endpoint. Quality of life (QoL) endpoints included changes from baseline to end-of-study in European Organisation for Research and Treatment of Cancer Quality-of-Life Questionnaire Core 30 (EORTC QLQ-C30) scores. Response according to phosphatase tensin homolog (PTEN) and MSS status were exploratory endpoints.

### 2.3. Assessments

Baseline computerized tomography (CT) or magnetic resonance imaging (MRI) scans of the chest, abdomen, and pelvis were obtained within 4 weeks of the first dose, then every 2 cycles from cycles 2–8 and every 3 cycles thereafter. Partial responses were confirmed by rescanning patients ~4 weeks after the previous scan. Patients attended an end-of-treatment (EOT) visit 30 to 40 days after receiving their last dose of study treatment, or before the start of subsequent anticancer therapy, if that was required sooner than 30 days after EOT for reasons other than disease progression, at which time patients entered post-treatment follow-up for PFS and OS. For patients who discontinued for reasons other than disease progression, CT or MRI scans were completed every 2 months for the first 6 months after the EOT visit, then every 3 months until disease progression. After disease progression, patients were followed for OS every 3 months. Toxicity was evaluated according to the National Cancer Institute Common Technology Criteria for Adverse Events, version 4.03. Patient reported outcomes were evaluated using EORTC QLQ-C30, at baseline, on day 1 of each cycle, and at EOT.

### 2.4. Biomarkers

Tumor specimens provided at study entry were studied for baseline biomarkers. *PTEN* mutation status was assessed by immunohistochemistry (IHC) with the monoclonal antibody (mAb) 6H2.1 Clone (Agilent/Dako) with a positive cut-off of >5% of tumor cells. MSI/deficient mismatch repair (dMMR) was assessed by IHC with a cut-off of <5% of tumor cells carrying any of the following MMR proteins: MLH1, MSH2, MSH6, and PMS2 (stained with Biocare mouse mAbs G168–15 [MLH-1] and FE11 [MSH2] and Epitomics rabbit mAbs EP49 [MSH6] and EP51 [PMS2]) or by nucleotide gain on mononucleotide markers (BAT-25, BAT-26, NR-21, NR-24 and MONO-27) using the Promega MSI Analysis System. Molecular profiling of 1100 genes was determined using a custom-designed Illumina Hybrid Selection Takeda/Broad Cancer Panel v2 (5.7 Mb) with tumor and matched-normal blood, at 500× and 250× coverage respectively. Associations between gene mutations and PFS length were analyzed at the gene level using a regression model, and at the pathway level using Fisher’s exact test.

### 2.5. Statistical analysis

Two interim analyses using the Bayesian predictive probability design with early stopping rules for futility were planned for the sapanisertib and sapanisertib + TAK-117 arms [22]. Arms were closed to enrollment if ≤6 patients from the first 20 patients treated (≥4 cycles of treatment), or ≤10 patients from the first 30 patients treated (≥4 cycles of treatment), achieved clinical benefit at 16 weeks in each arm.

The primary comparison of the primary endpoint of PFS was conducted between the paclitaxel and paclitaxel + sapanisertib treatment arms. Assuming an increase in median PFS from 4 months with paclitaxel alone to 6.5 months with sapanisertib in combination with paclitaxel (hazard ratio [HR] 0.615; approximately 38% reduction in the hazard rate), a total of 134 PFS events and 90 patients per treatment arm were required. This calculation is based on 80% power using a two-sided alpha of 5% and assuming a dropout rate of 15%. The intent-to-treat (ITT) population was defined as all randomized patients and was used for all time-to-event analyses, including the primary endpoint; distributions were estimated using Kaplan–Meier methodology. HRs along with two-sided 95% confidence intervals (CIs) were estimated using a stratified Cox regression model adjusted for histological subtype, lines of prior chemotherapy and prior taxane therapy. Response-evaluable and safety populations are described in the Supplementary Methods. No statistical adjustments were made for multiple comparisons. All statistical analyses were conducted using SAS Version 9.4.

## 3. Results

### 3.1. Patients

**Fig. 1.**
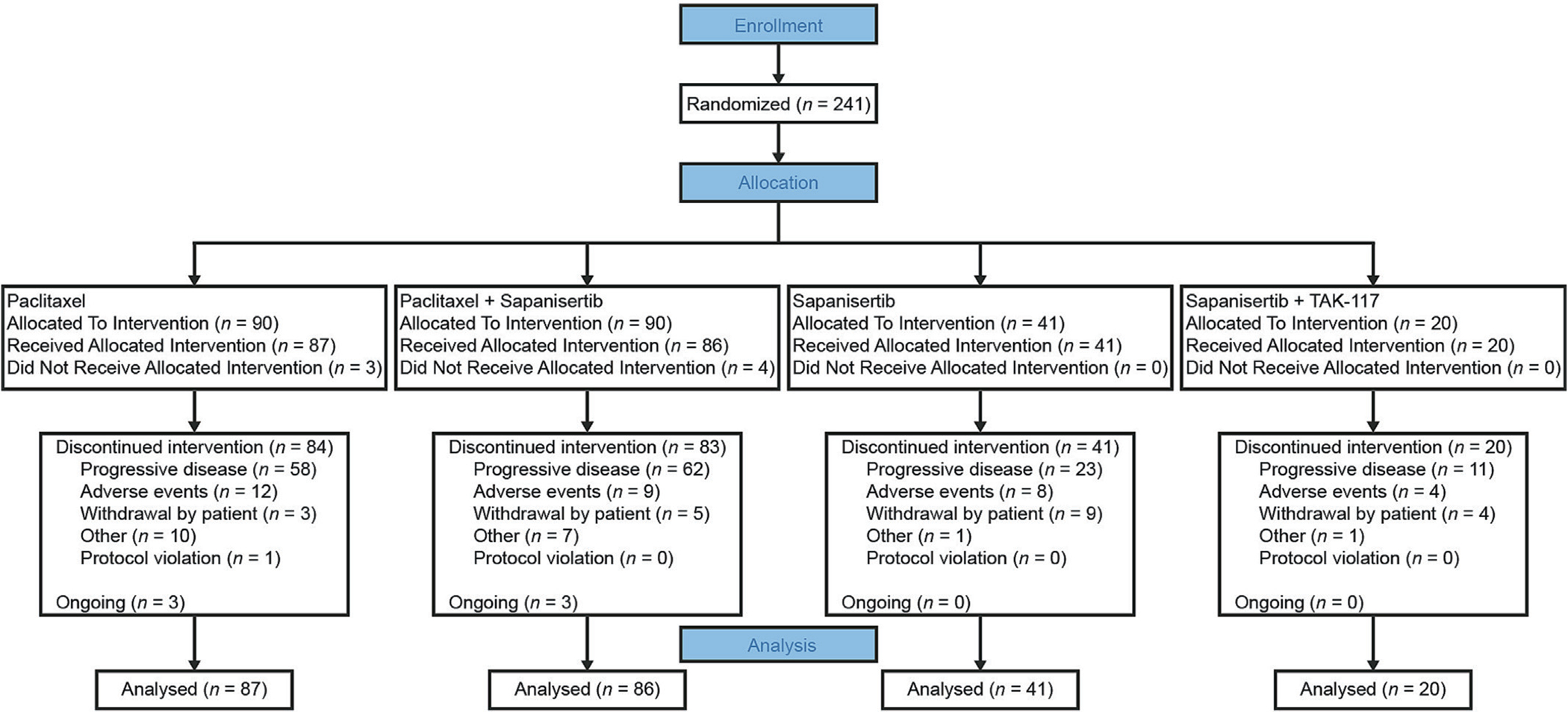
Patient disposition CONSORT diagram.

From September 2016 to October 2018, 241 patients from 60 sites in 10 countries were randomized to receive paclitaxel (*n* = 90), paclitaxel + sapanisertib (*n* = 90), single-agent sapanisertib (*n* = 41) or sapanisertib + TAK-117 (*n* = 20) (Fig. 1; sapanisertib and sapanisertib + TAK-117 arms were closed to enrolment after futility analyses). Patient baseline demographics and disease characteristics were well balanced (Table 1); median age was 64 (range 41–82) years, most patients had endometrioid adenocarcinoma (63.9%), 46.4% were disease stage IV and 51.1% and 46.8% had a baseline Eastern Cooperative Oncology Group performance status of 0 and 1, respectively. Overall, 46.1% and 37.8% of patients had received one or two lines of any prior therapy, respectively, including non-chemotherapy, surgery, and radiotherapy with most receiving carboplatin (98.3%) and paclitaxel (96.7%; not as a weekly regimen) previously.

### 3.2. Efficacy

**Fig. 2.**
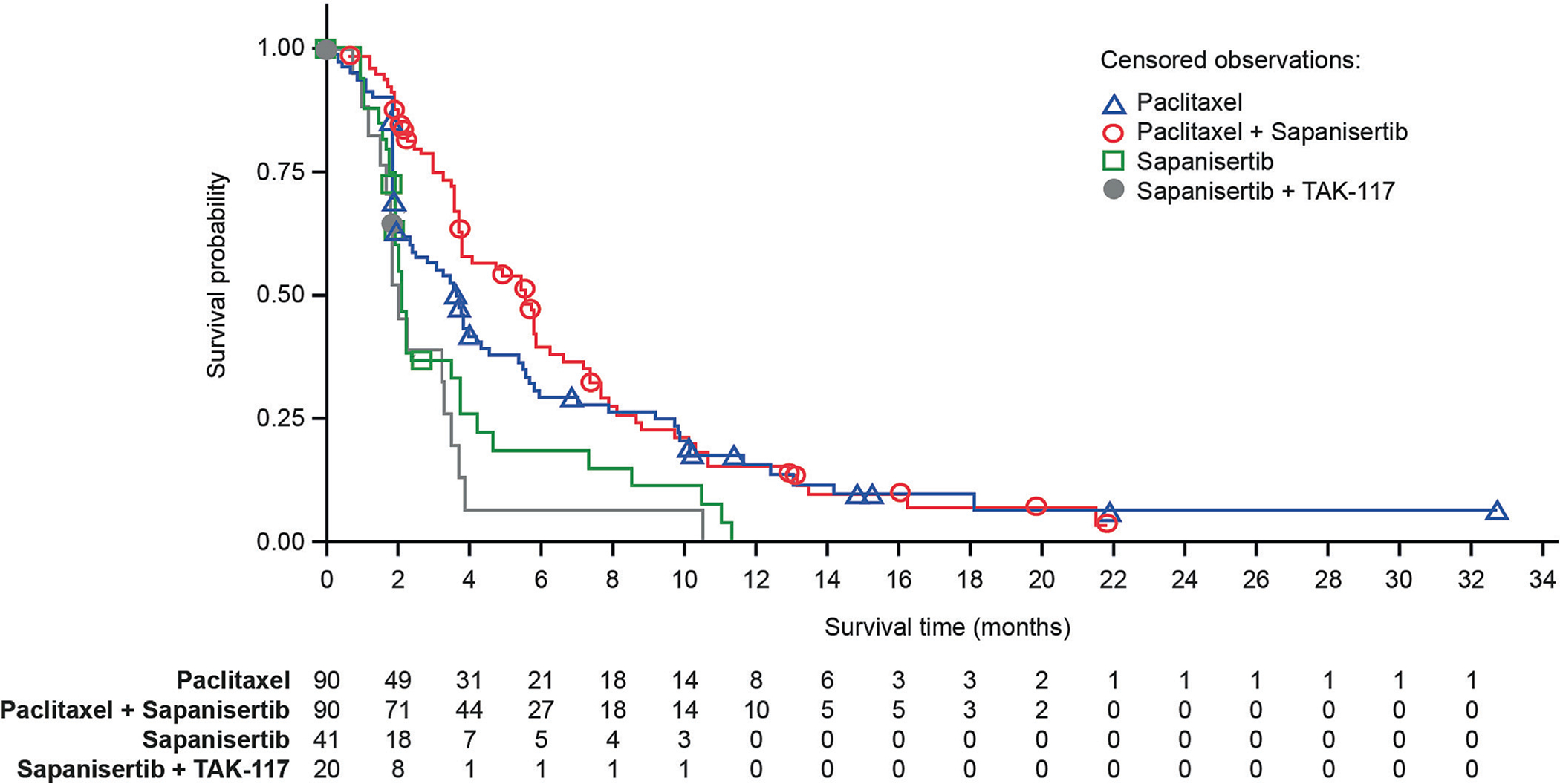
Progression-free survival in the intent-to-treat population.

After a median follow-up of 14.4 months (paclitaxel) or 17.2 months (paclitaxel + sapanisertib), median PFS was shorter with paclitaxel versus paclitaxel + sapanisertib (3.7 vs 5.6 months; HR 0.82; 95% CI 0.58–1.15; *p* = 0.139) (Fig. 2); however, the difference in PFS was not statistically significant, and the primary endpoint was not met. Median PFS for sapanisertib alone and sapanisertib + TAK-117 was 2.1 and 2.0 months, respectively. In patients with endometrioid histology, median PFS was 3.3 months with paclitaxel (*n* = 57) versus 5.7 months with paclitaxel + sapanisertib (*n* = 59) (HR 0.66; 95% CI 0.43–1.03) (Supplementary Fig. 1). In patients with non-endometrioid histology, median PFS was 5.4 with paclitaxel (*n* = 33) versus 3.6 months with paclitaxel + sapanisertib (*n* = 31) (HR 1.09; 95% CI 0.62–1.90) (Supplementary Fig. 1). Median OS in the ITT population was 14.6 months with paclitaxel versus 13.7 months with paclitaxel + sapanisertib (HR 1.01; 95% CI 0.67–1.53; *p* = 0.954) (Supplementary Fig. 2). Subgroup analysis showed median OS of 11.6 with paclitaxel versus 15.2 months with paclitaxel + sapanisertib (HR 0.83; 95% CI 0.50–1.38) in endometrioid patients, and 20.8 with paclitaxel versus 9.9 months with paclitaxel + sapanisertib (HR 1.59; 95% CI 0.78–3.29) in non-endometrioid patients. Subsequent anticancer therapies were received by 62.2% and 51.1% of patients in the paclitaxel and paclitaxel + sapanisertib arms, respectively. The most frequent therapies were doxorubicin, carboplatin, paclitaxel, and gemcitabine.

Response was assessed in all patients who received one dose of study drug (safety population; paclitaxel [*n* = 87], paclitaxel + sapanisertib [*n* = 86]) (Table 2). Confirmed ORR was 18.4% with paclitaxel (complete response [CR], *n* = 2; partial response [PR], *n* = 14) versus 24.4% (CR, *n* = 2; PR, *n* = 19) with paclitaxel + sapanisertib; CBR was 57.5% versus 80.2%; CBR-16 was 36.8% versus 51.2%. Response rates by patient histological subtype, *PTEN* mutation status, and MSI status are shown in Table 3. In patients with endometrioid histology, CBR was 55% with paclitaxel alone versus 84% with paclitaxel + sapanisertib; and in patients with non-endometrioid histology CBR was 63% versus 72%. No patients with high MSI status responded to paclitaxel + sapanisertib, compared with 15 with MSS status; similar results were observed with paclitaxel where only one patient with high MSI status had a response compared with 15 with MSS status. In patients receiving single-agent sapanisertib and sapanisertib + TAK-117, response was assessed at the planned interim analyses after 4 cycles of treatment. CBR-16 was 17.1% with sapanisertib and 5.0% with sapanisertib + TAK-117, and both arms were closed to enrollment per protocol.

### 3.3. Genomic analysis

Molecular profiling in a subgroup of 67 patients in this study confirmed that mutations in *PIK3CA* and *PTEN* were more common in endometrioid than non-endometrioid tumors, but were not associated with improved PFS with paclitaxel + sapanisertib compared with paclitaxel in patients with endometrioid histology, consistent with findings from other studies. However, mutations in the beta-catenin signaling pathway were associated with improved PFS with paclitaxel + sapanisertib compared with paclitaxel in patients with endometrioid histology (*p* < 0.05; Supplementary Tables 1–3; Supplementary Fig. 3).

### 3.4. Safety

Patients received a median of 4 cycles of paclitaxel (range 1–37), 5 cycles of paclitaxel + sapanisertib (range sapanisertib 1–23; paclitaxel 1–18), 2 cycles of single-agent sapanisertib and 2 cycles of sapanisertib + TAK-117. The most common reason for discontinuation of study drug was progressive disease (65.8%).

The most frequently reported (≥20% in any treatment arm) TEAEs of any grade with paclitaxel versus paclitaxel + sapanisertib included nausea (33.3% vs 60.5%), anemia (36.8% vs 55.8%), diarrhea (35.6% vs 55.8%), fatigue (44.8% vs 46.5%), decreased appetite (18.4% vs 38.4%), and alopecia (35.6% vs 31.4%) (Table 4). Grade ≥ 3 TEAE rates were 54.0% with paclitaxel versus 89.5% paclitaxel + sapanisertib.

Grade ≥ 3 TEAEs were experienced by 54.0% of patients in the paclitaxel arm, 89.5% in the paclitaxel + sapanisertib arm, 68.3% in the sapanisertib arm, and 70.0% in the sapanisertib + TAK-117 arm. The most frequently reported (≥2% in any treatment arm) grade ≥ 3 TEAEs with paclitaxel versus paclitaxel + sapanisertib included anemia (11.5% vs 20.9%), fatigue (4.6% vs 11.6%), neutropenia (3.4% vs 11.6%), hypophosphatemia (1.1% vs 11.6%), and pulmonary embolism (3.4% vs 10.5%) (Table 4). Grade ≥ 3 treatment-related TEAEs were reported in 24.1% of patients receiving paclitaxel, 66.3% receiving paclitaxel + sapanisertib, 43.9% receiving sapanisertib, and 60.0% receiving sapanisertib + TAK-117 (Supplementary Table 4).

Forty patients discontinued treatment due to TEAEs (paclitaxel, *n* = 12; paclitaxel + sapanisertib, *n* = 13; sapanisertib, *n* = 9; sapanisertib + TAK-117, *n* = 6). There were 15 on-study deaths that occurred between the first dose of study drug and 30 days after the last dose; four with paclitaxel, eight with paclitaxel + sapanisertib, one with sapanisertib and two with sapanisertib + TAK-117. Thirteen were considered related to the disease under study. The remaining two were due to hyperbilirubinemia (paclitaxel + sapanisertib) and general physical health deterioration (sapanisertib + TAK-117), both considered unrelated to study drug.

### 3.5. Patient-reported outcomes

In the ITT population, EORTC QLQ-C30 compliance was 96.1% with paclitaxel, and 93.6% with paclitaxel + sapanisertib. Mean global health status/QoL scores tended to show deterioration from baseline with both paclitaxel and paclitaxel + sapanisertib. The difference between mean change from baseline global health status/QoL scores for paclitaxel and paclitaxel + sapanisertib was <10 points (Supplementary Fig. 4) and not significant (*p* > 0.05). Median time to deterioration of global QoL was 2.1 months for both treatment arms.

## 4. Discussion

In this randomized phase 2 study, there was no statistically significant difference in PFS with paclitaxel + sapanisertib versus paclitaxel alone, and the primary endpoint was not met. Median PFS reported with paclitaxel (3.7 months) was as expected for this pre-treated population [23]. The addition of sapanisertib increased median PFS to 5.6 months, consistent with previously reported outcomes with other mTOR inhibitors and immunotherapeutic combinations in similar patient populations [24–26]. Median PFS values reported with the chemotherapy-free treatment arms of sapanisertib and sapanisertib + TAK-117 were inferior to the treatment arms containing paclitaxel.

Although chemotherapeutic treatment options for advanced endometrial cancer remain limited, there have been significant advances in immunotherapy in this setting in the last five years. Pembrolizumab, a programmed death receptor-1 (PD-1)-blocking antibody, has been approved since 2020 as monotherapy for the treatment of patients with advanced endometrial carcinoma that is MSI-high or dMMR, who have progressed after prior systemic therapy, and are not candidates for curative surgery or radiation [27–29]. Pembrolizumab in combination with lenvatinib has also been approved since 2020 for patients with endometrial cancer that is not MSI-high or dMMR, who have progressed after prior systemic therapy, and are not candidates for curative surgery or radiation [29,30]. Furthermore, pembrolizumab in combination with carboplatin-paclitaxel (NRG-GY018) resulted in longer progression-free survival than with carboplatin-paclitaxel alone in patients with advanced or recurrent endometrial cancer [31]. Another anti-PD-1 antibody, dostarlimab, has been approved since 2021 for the treatment of dMMR recurrent or advanced endometrial cancer that has progressed on or following a prior platinum-containing regimen [32]. Dostarlimab plus carboplatin-paclitaxel has recently been shown to improve PFS, compared to placebo, among patients with primary advanced or recurrent endometrial cancer, particularly in the dMMR and MSI-high population [33]. As treatment for advanced and recurrent endometrial cancer shifts towards combined chemotherapy and immunotherapy, additional combination strategies, such as with mTOR inhibitors may be worth investigating.

Single-agent temsirolimus mTOR inhibition previously demonstrated modest activity in advanced/recurrent endometrial carcinoma, although durable disease stabilization was observed in some patients. In our study, the ORR was lower with paclitaxel alone (18.4%) versus paclitaxel + sapanisertib (24.4%) and less improvement was observed in CBR (57.5% vs 80.2%, respectively) and CBR-16 (36.8% vs 51.2%, respectively). The ORR of 24.4% for patients treated with paclitaxel + sapanisertib was consistent with the ORR of 18% reported in a phase 1 study of sapanisertib in combination with paclitaxel in patients with advanced solid tumors, and the CBR was considerably higher (80% vs 50%) [34]. The ORR was also consistent with that reported with other mTOR inhibitors and immunotherapeutic combinations in similar pre-treated populations.

Subgroup analyses based on endometrioid histology were performed to assess differential response to paclitaxel versus paclitaxel + sapanisertib based on histologic subtype. The trend for improvement in PFS with the addition of sapanisertib to paclitaxel appeared to be driven by longer PFS in patients with endometrioid histology (HR 0.66; 95% CI 0.43–1.03) while there was no apparent benefit in patients with non-endometrioid histology (HR 1.09; 95% CI 0.62–1.90). Improvements in CBR and CBR-16 with paclitaxel + sapanisertib versus paclitaxel were also numerically greater in the endometrioid than non-endometrioid subgroup. Endometrioid histology is typically associated with a better prognosis and more mutations affecting the PI3K/AKT/mTOR pathway [9–11], which may explain why this subgroup was particularly responsive to inhibition of this pathway by sapanisertib. Molecular profiling in a subgroup of 67 patients in this study confirmed that mutations in *PIK3CA* and *PTEN* were more common in endometrioid than non-endometrioid tumors, but were not associated with improved PFS with paclitaxel + sapanisertib compared with paclitaxel in patients with endometrioid histology; however, mutations in the beta-catenin signaling pathway were associated with improved PFS with paclitaxel + sapanisertib compared with paclitaxel in patients with endometrioid histology. Further translational studies are needed to confirm these observations.

Patients with MSS status appeared to have a higher ORR versus patients with high MSI status overall, but there were no apparent differences according to whether patients received paclitaxel or paclitaxel in combination with sapanisertib. Although there were no treatment effects observed in this study according to MSS status, it is important to analyze clinical trial data according to different patient subgroups given the heterogeneity of this disease and the approval of therapies for specific subtypes. Immunotherapy has shown promise in patients with MSI-high endometrial cancer; single-agent pembrolizumab has recently received regulatory approval for patients with advanced endometrial cancer that is MSI-high or mismatch repair–deficient [35]. However, there is still an unmet need given the modest activity observed in patients with MSS status (response rates ranging from 3%–23%) [36]. The frequency of alterations in the PI3K/AKT/mTOR pathway across all histologies supports rational approaches to further develop this drug pathway.

The incidence of grade ≥ 3 TEAEs was higher with paclitaxel + sapanisertib versus paclitaxel, but toxicity with this combination was limited, with no new safety signals or notable differences in QoL. The most frequently reported grade ≥ 3 TEAEs included anemia, neutropenia, fatigue, and hypophosphatemia, which is consistent with the known safety profiles, and as expected for inhibition of the PI3K/AKT/mTOR pathway [23,34,37,38].

The primary endpoint of this study was not met and treatment with paclitaxel + sapanisertib did not improve outcomes. The trend for improvement in PFS of combination therapy was driven by outcomes in patients with endometrioid subtype tumors, who demonstrated greater numerical improvements in PFS, CBR, and CBR-16 with paclitaxel + sapanisertib versus paclitaxel alone than patients with non-endometrioid histology. The incidence of grade ≥ 3 TEAEs was higher in patients receiving paclitaxel + sapanisertib, but treatment with the combination had limited toxicity with no new safety signals. Given these encouraging preliminary data, future studies should investigate sapanisertib in combination with other agents in patients with endometrioid histology.

## Supplementary Material

1

## Figures and Tables

**Table 1:** Patient baseline demographics and disease characteristics.

ITT Population	Paclitaxel (*n* = 90)	Paclitaxel + Sapanisertib (*n* = 90)	Sapanisertib (*n* = 41)	Sapanisertib + TAK-117 (*n* = 20)

Median age, years (range)	65.0 (41–80)	64.5 (45–82)	64.0 (46–76)	62.0 (41–80)
Race, *n* (%)				
White	77 (85.6)	78 (86.7)	37 (90.2)	18 (90.0)
Asian	6 (6.7)	3 (3.3)	1 (2.4)	1 (5.0)
Other	4 (4.4)	6 (6.7)	3 (7.3)	1 (5.0)
Not reported	3 (3.3)	3 (3.3)	0	0
Histological classification^a^, *n* (%)				
Endometrioid adenocarcinoma, NOS	54 (60.0)	59 (65.6)	25 (61.0)	16 (80.0)
Serous cystadenocarcinoma, NOS	18 (20.0)	19 (21.1)	8 (19.5)	0
Mixed cell adenocarcinoma	5 (5.6)	3 (3.3)	2 (4.9)	1 (5.0)
Clear cell adenocarcinoma, NOS	4 (4.4)	1 (1.1)	0	0
Carcinosarcoma, NOS	8 (8.9)	8 (8.9)	6 (14.6)	3 (15.0)
Unknown	1 (1.1)	0	0	0
Histological grade^a^, *n* (%)				
Well differentiated (G1)	18 (20.9)	16 (18.8)	7 (17.5)	3 (15.8)
Moderatelydifferentiated (G2)	19 (22.1)	18 (21.2)	13 (32.5)	5 (26.3)
Poorly differentiated (G3)	42 (48.8)	43 (50.6)	17 (42.5)	8 (42.1)
Undifferentiated (G4)	7 (8.1)	8 (9.4)	3 (7.5)	3 (15.8)
Missing	4	5	1	1
Disease stage^b^, *n* (%)				
I	15 (17.2)	8 (8.9)	7 (17.5)	1 (5.0)
II	1 (1.1)	0	0	0
III	19 (21.8)	30 (33.3)	9 (22.5)	2 (10.0)
IV	36 (41.4)	40 (44.4)	19 (47.5)	15 (75.0)
Unknown	15 (17.2)	10 (11.1)	5 (12.5)	1 (5.0)
Other	1 (1.1)	1 (1.1)	0	0
ECOG PS, n (%)				
0	41 (47.1)	49 (57.6)	22 (53.7)	7 (35.0)
1	44 (50.6)	35 (41.2)	18 (43.9)	12 (60.0)
2	2 (2.3)	1 (1.2)	1 (2.4)	1 (5.0)
Missing	3	5	0	0
Prior lines of therapy^c^, *n* (%)				
1	43 (47.8)	41 (45.6)	17 (41.5)	10 (50.0)
2	35 (38.9)	29 (32.2)	19 (46.3)	8 (40.0)
3	11 (12.2)	18 (20.0)	5 (12.2)	1 (5.0)
4	1 (1.1)	2 (2.2)	0	1 (5.0)
Prior lines of chemotherapy, *n* (%)				
1	51 (56.7)	49 (54.4)	21 (51.2)	11 (55.0)
2	39 (43.3)	41 (45.6)	20 (48.8)	9 (45.0)
Prior taxane therapy, *n* (%)				
Yes	86 (95.6)	87 (96.7)	39 (95.1)	20 (100)
No	4 (4.4)	3 (3.3)	2 (4.9)	0

Abbreviations: ECOG PS, Eastern Cooperative Oncology Group performance status; G, grade; ITT, intent-to-treat; NOS, not otherwise specified.

a At initial diagnosis.

b According to patient’s record prior to study entry.

c Includes surgery, radiotherapy and non-chemotherapeutic drugs.

**Table 2:** Confirmed overall response rate in the safety population.

Safety Population, *n* (%)	Paclitaxel (*n* = 87)	Paclitaxel + Sapanisertib (*n* = 86)	Sapanisertib (*n* = 41)	Sapanisertib + TAK-117 (*n* = 20)

CR	2 (2.3)	2 (2.3)	0	0
PR	14 (16.1)	19 (22.1)	2 (4.9)	0
SD	34 (39.1)	48 (55.8)	12 (29.3)	7 (35.0)
SD ≥16 weeks	16 (18.4)	23 (26.7)	5 (12.2)	1 (5.0)
ORR	16 (18.4)	21 (24.4)	2 (4.9)	0
Odds ratio (95% CI), comparison versus paclitaxel	–	1.34 (0.64–2.81)	0.22 (0.04–1.19)	0.00
CBR	50 (57.5)	69 (80.2)	14 (34.1)	7 (35.0)
Odds ratio (95% CI), comparison versus paclitaxel	–	2.84 (1.43–5.63)	0.42 (0.19–0.92)	0.47 (0.16–1.37)
CBR-16	32 (36.8)	44 (51.2)	7 (17.1)	1 (5.0)
Odds ratio (95% CI), comparison versus paclitaxel	–	2.62 (0.89–7.67)	0.15 (0.05–0.51)	0.07 (0.01–0.67)

Abbreviations: CBR, clinical benefit rate; CBR-16, CBR at 16 weeks; CI, confidence interval; CR, complete response; ORR, overall response rate; PR, partial response; SD, stable disease.

**Table 3:** Overall response rate by histological subtype, PTEN mutation status and MSI status in the safety population.

Safety Population, *n* (%)	Paclitaxel (*n* = 87)	Paclitaxel + Sapanisertib (*n* = 86)	Paclitaxel (*n* = 87)	Paclitaxel + Sapanisertib (*n* = 86)

	Endometrioid		Non-endometrioid	
*Histological*	55	57	32	29
*subtype, n*				
ORR	9 (16)	13 (23)	7 (22)	8 (28)
CBR	30 (55)	48 (84)	20 (63)	21 (72)
CBR-16	17 (31)	32 (56)	15 (47)	12 (41)
	*PTEN* Negative		*PTEN* Positive	
*PTEN status*^a^ *n*	11	18	44	38
ORR	2 (18.2)	1 (5.6)	12 (27.3)	12 (31.5)
CBR	7 (63.6)	13 (72.2)	27 (61.4)	29 (76.3)
CBR-16	5 (45.5)	6 (33.3)	22 (50.0)	21 (55.3)
	MSS		MSI	
*MSI status,*^b^ *n*	57	54	9	10
ORR	14 (24.6)	15 (27.8)	1 (11.1)	0
CBR	33 (57.9)	40 (74.1)	8 (88.9)	8 (80.0)
CBR-16	25 (43.39)	27 (50.0)	4 (44.4)	4 (40.0)
	MSS and Endometrioid		MSS and Non-endometrioid	
*MSS and histological subtype, n*	33	31	24	23
ORR	7 (21.2)	8 (25.8)	7 (29.2)	7 (30.4)
CBR	18 (54.6)	24 (77.4)	15 (62.5)	16 (69.6)
CBR-16	12 (36.4)	16 (51.6)	13 (54.2)	11 (47.8)

Abbreviations: CBR, clinical benefit rate; CBR-16, CBR at 16 weeks; dMMR, deficient mismatch repair; IHC, immunohistochemistry; MMR, mismatch repair; MSI, microsatellite instability; MSS, microsatellite stable; ORR, overall response rate; PCR, polymerase chain reaction; PTEN, phosphatase and tensin homolog.

a PTEN IHC positive cut-off >5% tumor cells.

b MSI/dMMR defined as IHC <5% tumor cells of any MMR proteins or nucleotide gain by PCR.

**Table 4:** Most frequent (≥20% any treatment arm) any grade treatment-emergent adverse events and most frequent (≥2% of patients overall) grade ≥ 3 treatment-emergent adverse events by preferred term in the safety population.

Safety Population, *n* (%)	Paclitaxel (*n* = 87)	Paclitaxel + Sapanisertib (*n* = 86)	Sapanisertib (*n* = 41)	Sapanisertib + TAK-117 (*n* = 20)

Any grade TEAEs	87 (100)	86 (100)	41 (100)	20 (100)
Nausea	29 (33.3)	52 (60.5)	30 (73.2)	16 (80.0)
Diarrhea	31 (35.6)	48 (55.8)	15 (36.6)	13 (65.0)
Fatigue	39 (44.8)	40 (46.5)	18 (43.9)	7 (35.0)
Anemia	32 (36.8)	48 (55.8)	5 (12.2)	6 (30.0)
Vomiting	20 (23.0)	24 (27.9)	31 (75.6)	15 (75.0)
Decreased appetite	16 (18.4)	33 (38.4)	20 (48.8)	8 (40.0)
Constipation	25 (28.7)	20 (23.3)	14 (34.1)	5 (25.0)
Alopecia	31 (35.6)	27 (31.4)	1 (2.4)	0
Asthenia	7 (8.0)	25 (29.1)	9 (22.0)	10 (50.0)
Dyspnea	18 (20.7)	24 (27.9)	6 (14.6)	3 (15.0)
Cough	21 (24.1)	19 (22.1)	8 (19.5)	1 (5.0)
Abdominal pain	13 (14.9)	22 (25.6)	6 (14.6)	4 (20.0)
Hyperglycemia	8 (9.2)	17 (19.8)	15 (36.6)	5 (25.0)
Hypomagnesemia	11 (12.6)	19 (22.1)	7 (17.1)	1 (5.0)
Stomatitis	4 (4.6)	22 (25.6)	10 (24.4)	2 (10.0)
Peripheral neuropathy	12 (13.8)	22 (25.6)	3 (7.3)	0
Urinary tract infection	9 (10.3)	18 (20.9)	6 (14.6)	3 (15.0)
Pyrexia	12 (13.8)	13 (15.1)	5 (12.2)	4 (20.0)
Arthralgia	11 (12.6)	21 (24.4)	0	0
Neutropenia	10 (11.5)	19 (22.1)	1 (2.4)	1 (5.0)
Peripheral edema	18 (20.7)	9 (10.5)	2 (4.9)	1 (5.0)
Upper abdominal pain	6 (6.9)	13 (15.1)	4 (9.8)	4 (20.0)
Increased alanine aminotransferase	5 (5.7)	7 (8.1)	1 (2.4)	6 (30.0)
Increased aspartate aminotransferase	3 (3.4)	7 (8.1)	2 (4.9)	6 (30.0)
Increased blood creatinine	3 (3.4)	6 (7.0)	3 (7.3)	4 (20.0)
Grade ≥3 TEAEs	47 (54.0)	77 (89.5)	28 (68.3)	14 (70.0)
Nausea	2 (2.3)	3 (3.5)	5 (12.2)	6 (30.0)
Diarrhea	4 (4.6)	8 (9.3)	1 (2.4)	2 (10.0)
Vomiting	2 (2.3)	2 (2.3)	4 (9.8)	6 (30.0)
Stomatitis	0	1 (1.2)	6 (14.6)	0
Abdominal pain	3 (3.4)	1 (1.2)	1 (2.4)	1 (5.0)
Anemia	10 (11.5)	18 (20.9)	1 (2.4)	4 (20.0)
Neutropenia	3 (3.4)	10 (11.6)	0	0
Leukopenia	2 (2.3)	8 (9.3)	0	0
Fatigue	4 (4.6)	10 (11.6)	6 (14.6)	1 (5.0)
Asthenia	1 (1.1)	1 (1.2)	3 (7.3)	5 (25.0)
General physical health deterioration	3 (3.4)	4 (4.7)	1 (2.4)	2 (10.0)
Hypophosphatemia	1 (1.1)	10 (11.6)	1 (2.4)	0
Hyperglycemia	1 (1.1)	2 (2.3)	6 (14.6)	0
Decreased appetite	0	2 (2.3)	4 (9.8)	1 (5.0)
Dehydration	0	2 (2.3)	1 (2.4)	2 (10.0)

Abbreviations: TEAEs, treatment-emergent adverse events.

## Data Availability

The datasets, including the redacted study protocol, redacted statistical analysis plan, and individual participant data supporting the results reported in this article, will be made available within three months from initial request to researchers who provide a methodologically sound proposal. The data will be provided after their de-identification, in compliance with applicable privacy laws, data protection and requirements for consent and anonymization.

## HIGHLIGHTS

- Patients with endometrioid tumors showed improvement (PFS, CBR, and CBR-16) with paclitaxel + sapanisertib vs paclitaxel.
- There were no significant differences in PFS between the paclitaxel and paclitaxel + sapanisertib arms.
- Treatment with paclitaxel + sapanisertib had limited toxicity with no new safety signals.
