# Supplementary material for: A randomized phase 2 study of sapanisertib in combination with paclitaxel versus paclitaxel alone in women with advanced, recurrent, or persistent endometrial cancer
Source: Gynecol Oncol. Author manuscript; Available in PMC 2024 May 17. (PMC11100409; doi:10.1016/j.ygyno.2023.09.013)
Supplement: 1 [file NIHMS1983828-supplement-1.docx]

**SUPPLEMENTARY APPENDIX**

**Title:** A randomized phase 2 study of sapanisertib in combination with paclitaxel versus paclitaxel alone in women with advanced, recurrent, or persistent endometrial cancer

**Authors:** Sileny N. Han, Amit Oza, Nicoletta Colombo, Ana Oaknin, Francesco Raspagliesi, Robert M. Wenham, Elena Ioana Braicu, Andrea Jewell, Vicky Makker, Jonathan Krell, Eva María Guerra Alía, Jean-François Baurain, Zhenqiang Su, Rachel Neuwirth, Sylvie Vincent, Farhad Sedarati, Douglas V. Faller, Giovanni Scambia

**SUPPLEMENTARY METHODS**

**Inclusion Criteria**

- Histologic or cytologic diagnosis of endometrial carcinoma (including endometrioid, serous, mixed adenocarcinoma, clear-cell carcinoma, or carcinosarcoma).
- Evidence that the endometrial cancer was advanced, recurrent, or persistent and had relapsed or was refractory to curative therapy or established treatments.
- At least 1 prior platinum-based chemotherapeutic regimen, but not more than 2 prior chemotherapeutic regimens, for management of endometrial carcinoma.
  - Prior treatment may have included chemotherapy, chemotherapy/radiation therapy, and/or consolidation/maintenance therapy.
  - Chemotherapy administered in conjunction with primary radiation as a radiosensitized therapy was considered a systemic chemotherapy regimen.
- Measurable disease by Response Evaluation Criteria in Solid Tumors, version 1.1, defined as at least 1 lesion that could be accurately measured in at least 1 dimension (longest diameter was recorded).
  - Each lesion must have been ≥10 mm in long axis when measured by computerized tomography (CT), magnetic resonance imaging (MRI), or caliper measurement by clinical exam.
  - Lymph nodes must have been ≥15 mm in short axis when measured by CT or MRI.
- Tumor accessible and patient consented to undergo fresh tumor biopsies.
- Female patients 18 years or older.
- Eastern Cooperative Oncology Group performance status of 0 to 2.
- Female patients who:
- Were postmenopausal for at least 1 year before the screening visit, OR
- Were surgically sterile, OR
- If they were of childbearing potential, agreed to practice one highly effective method of contraception and one additional effective (barrier) method at the same time, from the time of signing the informed consent through 90 days (or longer, as mandated by local labelling, e.g., United States Prescribing Information, Summary of Product Characteristics) after the last dose of study drug, OR
- Agreed to practice true abstinence when this was in line with the preferred and usual lifestyle of the subject (periodic abstinence [e.g., calendar, ovulation, symptothermal, postovulation methods], withdrawal, spermicides only, and lactational amenorrhea were not acceptable methods of contraception. Female and male condoms should not have been used together).
- Clinical laboratory values as specified below within 4 weeks before the first dose of study drug:
- Bone marrow reserve consistent with absolute neutrophil count ≥1500/µL; platelet count ≥100,000/µL; glycosylated hemoglobin (HbA1c) <6.5%.
- Total bilirubin must be ≤1.5 times the upper limit of the normal range (ULN).
- Alanine aminotransferase (ALT) or aspartate aminotransferase (AST) must be ≤2.5 × ULN. AST and ALT may be elevated up to 5 × ULN if their elevation can be reasonably ascribed to the presence of metastatic disease in the liver.
- Creatinine clearance ≥50 mL/min/1.73 m^2^, based either on Cockcroft-Gault estimate or on a 12- or 24-hour urine collection.
- Fasting serum glucose <130 mg/dL and fasting triglycerides ≤300 mg/dL.
- Ability to swallow oral medications, willingness to perform mucositis prophylaxis, and suitable venous access for the study-required blood sampling.

Voluntary written consent had to be given before performance of any study-related procedure not part of standard medical care, with the understanding that consent may have been withdrawn by the patient at any time without prejudice to future medical care.

**Exclusion Criteria**

- Positive serum pregnancy test during the screening period or a positive urine pregnancy test on day 1 before first dose of study drug.
  - Women who were lactating and breastfeeding were not eligible.
- Previous treatment with any weekly taxane regimen.
- History of severe hypersensitivity reactions to paclitaxel or any of its excipients.
- Previous treatment with phosphoinositide-3 kinase (PI3K), protein kinase B, dual PI3K/mammalian target of rapamycin inhibitors, TORC1/2 inhibitors, or TORC1 inhibitors.
- Initiation of treatment with hematopoietic growth factors, transfusions of blood and blood products, or systemic corticosteroids (either intravenous [IV] or oral steroids, excluding inhalers) within 1 week before administration of the first dose of study drug (patients already receiving erythropoietin on a chronic basis for ≥4 weeks were eligible).
- Patients who were taking proton pump inhibitors (PPIs) within 7 days of the first dose of study drug or who required treatment with PPIs throughout the trial or those who were taking H_2_ receptor antagonists within 24 hours of the first dose of study drug.
- A prothrombin time or activated partial thromboplastin time above the ULN, or a history of a coagulopathy or bleeding disorder.
- Known hepatitis B surface antigen-positive, or known or suspected active hepatitis C infection.
- Sensory or motor neuropathy grade ≥2.
- Central nervous system (CNS) metastasis, endometrial leiomyosarcoma, or endometrial stromal sarcoma.
- Manifestations of malabsorption due to prior gastrointestinal surgery, gastrointestinal disease, or for some other reason that may alter the absorption of sapanisertib or TAK-117.
  - In addition, patients with enteric stomata were also excluded.
- Other clinically significant comorbidities, such as uncontrolled pulmonary disease, active CNS disease, active infection, or any other condition that could compromise participation of the patient in the study.
- Known HIV infection.
- History of any of the following within the last 6 months before administration of the first dose of study drug:
- Ischemic myocardial event, including angina requiring therapy and artery revascularization procedures.
- Ischemic cerebrovascular event, including transient ischemic attack and artery revascularization procedures.
- Requirement for inotropic support (excluding digoxin) or serious (uncontrolled) cardiac arrhythmia (including atrial flutter/fibrillation, ventricular fibrillation, or ventricular tachycardia).
- Placement of a pacemaker for control of rhythm.
- New York Heart Association class III or IV heart failure.
- Pulmonary embolism.
- Significant active cardiovascular or pulmonary disease before administration of the first dose of study drug, including:
- Uncontrolled hypertension (i.e., either systolic blood pressure >180 mm Hg or diastolic blood pressure >95 mm Hg).
- Pulmonary hypertension.
- Uncontrolled asthma or oxygen saturation <90% by arterial blood gas analysis or pulse oximetry on room air.
- Significant valvular disease, severe regurgitation or stenosis by imaging independent of symptom control with medical intervention, or history of valve replacement.
- Medically significant (symptomatic) bradycardia.
- History of arrhythmia requiring an implantable cardiac defibrillator.
- Baseline prolongation of the rate-corrected QT interval (QTc; e.g., repeated demonstration of QTc interval >480 msec, or history of congenital long QT syndrome, or torsades de pointes).
- Diagnosed or treated for another malignancy within 2 years before administration of the first dose of study drug or previously diagnosed with another malignancy with any evidence of residual disease. Patients with nonmelanoma skin cancer or carcinoma in situ of any type are not excluded if they have undergone complete resection. Patients with endometrioid histology and histologically confirmed expression of estrogen receptors and/or progesterone receptors who have not received prior endocrine therapy and for whom endocrine therapy is currently indicated.

**Assessments**

Patient reported outcomes were evaluated using the quality of life instrument European Organisation for Research and Treatment of Cancer Quality-of-Life Questionnaire Core 30 (EORTC QLQ-C30): a 30-item questionnaire incorporating 5 functional scales (physical, role, emotional, cognitive, and social functioning), 1 global health status scale, 3 symptom scales (fatigue, nausea/vomiting, pain), and 6 single items (dyspnea, insomnia, appetite loss, constipation, diarrhea, financial difficulties).

**Statistical Analyses – Patient Population**

The response-evaluable population comprised all patients who received ≥1 dose of study drug (safety population) and had measurable disease at baseline and 1 post-baseline disease assessment; this was used in the primary analyses of overall response rate (ORR), clinical benefit rate (CBR) and CBR at 16 weeks (CBR-16). The safety population was used in all safety analyses and the sensitivity analyses of ORR, CBR and CBR-16.

**SUPPLEMENTARY TABLES**

**Supplementary Table 1.** Association of progression-free survival and genes in all 67 patients with genomic data for molecular profiling.

| **Mutated Genes** | **Paclitaxel** | | **Paclitaxel +  Sapanisertib** | |
| --- | --- | --- | --- | --- |
|  | **Number of patients with mutation** | **Coefficient (*p*-value)** | **Number of patients with mutation** | **Coefficient (*p*-value)** |
| *DLC1* | 4 |  | 6 | -5.76 (0.0221) |
| *MXRA5* | 4 |  | 3 | 7.51 (0.0103) |
| *CTNNB1* | 3 |  | 4 | 6.34 (0.0141) |
| *BRIP1* | 4 | 9.07 (0.0309) | 3 | 7.58 (0.0191) |
| *SMC5* | 3 | 14.83 (0.0035) | 0 |  |
| *DICER1* | 3 | 13.85 (0.006) | 2 |  |
| *USP24* | 3 | 13.85 (0.006) | 1 |  |
| *GTF3C1* | 4 | 9.13 (0.0365) | 2 |  |

**Supplementary Table 2.** Association of progression-free survival and genes in 36 patients with endometrioid subtypes.

| **Mutated Genes** | **Paclitaxel** | | **Paclitaxel +  Sapanisertib** | |
| --- | --- | --- | --- | --- |
|  | **Number of patients with mutation** | **Coefficient (*p*-value)** | **Number of patients with mutation** | **Coefficient (*p*-value)** |
| *CTNNB1* | 1 |  | 4 | 6.3 (0.022) |
| *NAV3* | 2 |  | 3 | 8.2 (0.027) |
| *BRIP1* | 2 |  | 3 | 9.8 (0.006) |
| *DLC1* | 3 |  | 5 | -5.8 (0.036) |
| *PTEN* | 6 | 8.5 (0.046) | 8 |  |
| *MTOR* | 2 | 10.4 (0.029) | 5 |  |

**Supplementary Table 3.** Mutated pathways associated with progression-free survival in 36 patients with endometrioid subtypes.

| **Mutated Pathways** | **Odds Ratio for PFS >6 Months (*p*-value)** | |
| --- | --- | --- |
|  | **Paclitaxel** | **Paclitaxel +  Sapanisertib** |
| Androgen receptor activation and downstream signaling in prostate cancer |  | Inf (0.017) |
| B cell signaling in hematological malignancies |  | Inf (0.036) |
| Development_Negative regulation of WNT/beta-catenin signaling in the cytoplasm |  | Inf (0.044) |
| Development_WNT/beta-catenin signaling in the cytoplasm |  | 12.5 (0.045) |
| FGF signaling in gastric cancer |  | Inf (0.036) |
| Ligand-independent activation of androgen receptor in prostate cancer |  | Inf (0.013) |
| Main chemotherapy drugs and their action in SCLC cells |  | 14.7 (0.038) |
| Mechanisms of drug resistance in multiple myeloma |  | Inf (0.036) |
| Putative role of estrogen receptor and androgen receptor signaling in progression of lung cancer |  | 14.7 (0.038) |
| Regulation of beta-catenin activity in colorectal cancer |  | Inf (0.044) |
| Regulation of GSK3 beta in bipolar disorder |  | Inf (0.036) |
| Role of IL-8 in melanoma |  | Inf (0.009) |
| SHH signaling in colorectal cancer |  | 18.0 (0.020) |
| Signal transduction_angiotensin II/AGTR1 signaling via notch, beta-catenin and NF-kB pathways |  | 18.0 (0.020) |
| Stem cells_EGF-induced proliferation of type C cells in SVZ of adult brain |  | 14.7 (0.038) |
| TGF-beta 1-induced transactivation of membrane receptors signaling in HCC |  | 18.0 (0.020) |
| TGF-beta signaling via kinase cascades in breast cancer |  | Inf (0.036) |
| Transcription_androgen receptor nuclear signaling |  | 14.7 (0.038) |
| Translation_regulation of EIF2 activity |  | 14.7 (0.038) |
| Development_TGF-beta-dependent induction of EMT via MAPK | 36 (0.022) | Inf (0.036) |

Abbreviations: AGTR1, angiotensin II receptor Type I; EGF, epidermal growth factor; EIF2, eukaryotic initiation factor 2; EMT, epithelial-mesenchymal transition; FGF, fibroblast growth factor; GSK3, glycogen synthase kinase-3; HCC, hepatocellular carcinoma; IL-8, interleukin-8; Inf, infinity; MAPK, mitogen-activated protein kinase; NF-kB, nuclear factor kappa B; PFS, progression-free survival; SCLC, small-cell lung cancer; SHH, sonic hedgehog; SVZ, subventricular zone; TGF, transforming growth factor; WNT, wingless/integration.

**Supplementary Table 4.** Most frequent (≥2% of patients overall) grade ≥3 treatment-related TEAEs by preferred term in the safety population.

| **Safety Population, n (%)** | **Paclitaxel**  **(n = 87)** | **Paclitaxel + Sapanisertib**  **(n = 86)** | **Sapanisertib**  **(n = 41)** | **Sapanisertib +  TAK-117**  **(n = 20)** |
| --- | --- | --- | --- | --- |
| Grade ≥3 treatment-related TEAEs | 21 (24.1) | 57 (66.3) | 18 (43.9) | 12 (60.0) |
| Anemia | 5 (5.7) | 13 (15.1) | 1 (2.4) | 1 (5.0) |
| Neutropenia | 3 (3.4) | 10 (11.6) | 0 | 0 |
| Leukopenia | 0 | 8 (9.3) | 0 | 0 |
| Diarrhea | 3 (3.4) | 6 (7.0) | 1 (2.4) | 2 (10.0) |
| Nausea | 1 (1.1) | 0 | 5 (12.2) | 6 (30.0) |
| Vomiting | 1 (1.1) | 0 | 4 (9.8) | 6 (30.0) |
| Stomatitis | 0 | 1 (1.2) | 5 (12.2) | 0 |
| Fatigue | 1 (1.1) | 9 (10.5) | 4 (9.8) | 1 (5.0) |
| Asthenia | 0 | 1 (1.2) | 3 (7.3) | 5 (25.0) |
| Hyperglycemia | 0 | 2 (2.3) | 6 (14.6) | 0 |
| Hypophosphatemia | 0 | 5 (5.8) | 1 (2.4) | 0 |
| Decreased appetite | 0 | 1 (1.2) | 4 (9.8) | 0 |
| Peripheral neuropathy | 3 (3.4) | 4 (4.7) | 0 | 0 |

Abbreviations: TEAEs, treatment-emergent adverse events.

**SUPPLEMENTARY FIGURES**

**Supplementary Fig. 1.** Forest plot of progression-free survival by subgroup.

**
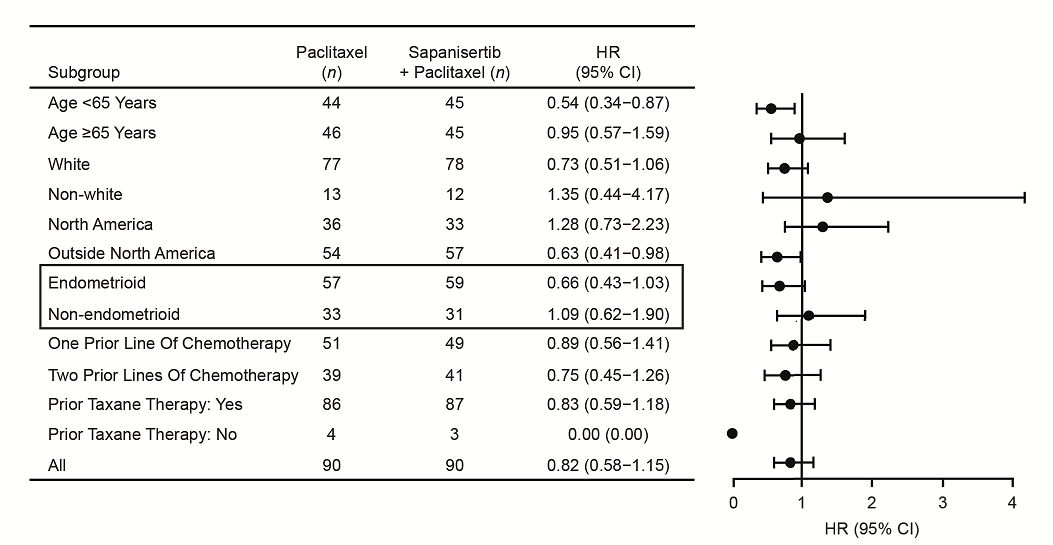
**

CI, confidence interval; HR, hazard ratio.

**Supplementary Fig. 2.** Kaplan–Meier analysis of overall survival in the intent-to-treat population.

**
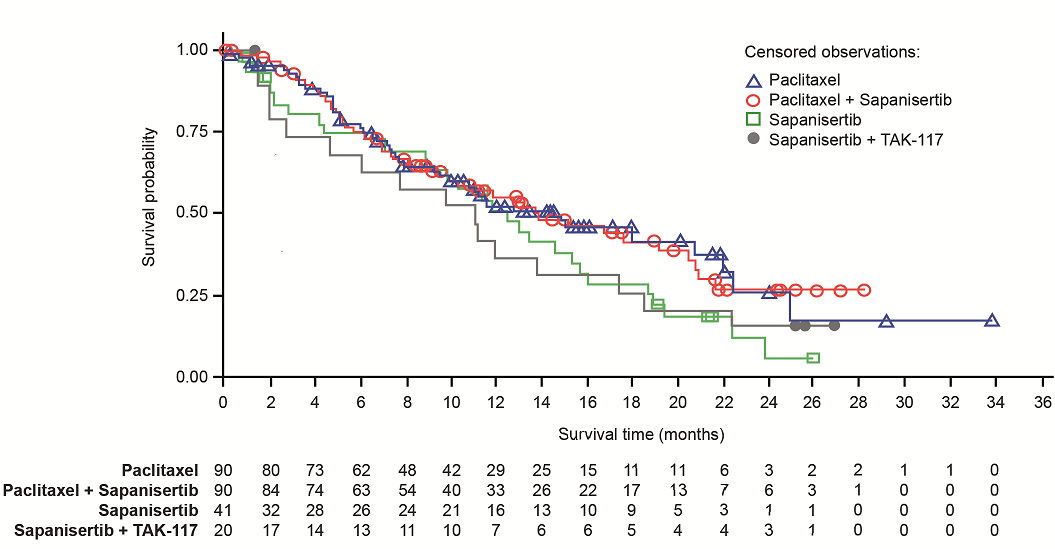
**

**Supplementary Fig. 3.** Frequency of mutated genes in patients with endometrioid (*n* = 42) and non-endometrioid (*n* = 25) histology with genomic data for molecular profiling.
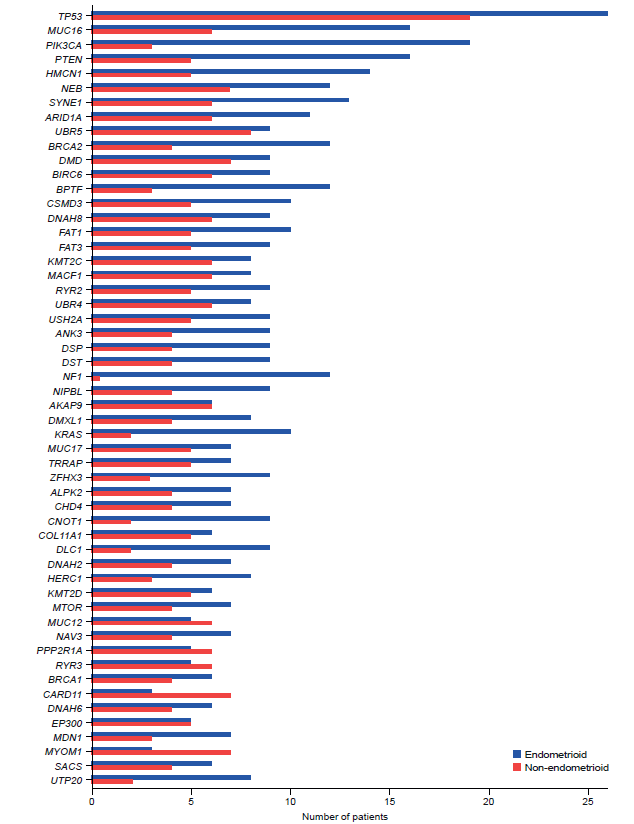


**Supplementary Fig. 4.** Mean change from baseline in European Organisation for Research and Treatment of Cancer Quality-of-Life Questionnaire Core 30 global health status/quality of life score over time in the intent-to-treat population.
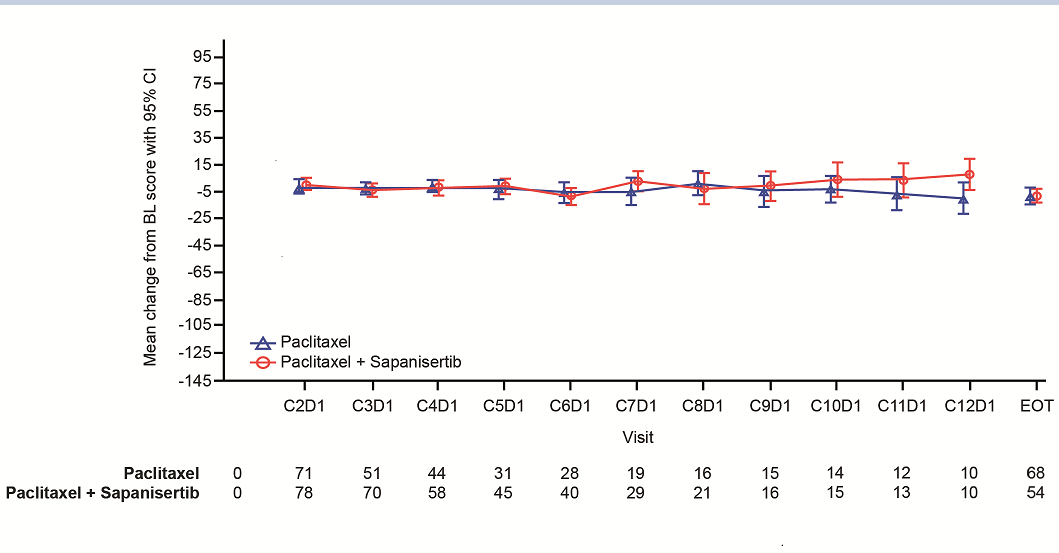


BL, baseline; C, cycle; CI, confidence interval; D, day; EOT, end of treatment.
